# Cognitive Control of Saccadic Eye Movements in Children with Developmental Coordination Disorder

**DOI:** 10.1371/journal.pone.0165380

**Published:** 2016-11-03

**Authors:** Claudia C. Gonzalez, Mark Mon-Williams, Siobhan Burke, Melanie R. Burke

**Affiliations:** 1 College of Health and Life Sciences, Sports, Health and Exercise Sciences, Brunel University London, Uxbridge, United Kingdom; 2 School of Psychology, Faculty of Medicine and Health, University of Leeds, Leeds, United Kingdom; 3 Norwich Medical School, University of East Anglia, Norwich, United Kingdom; Birkbeck College, UNITED KINGDOM

## Abstract

The ability to use advance information to prepare and execute a movement requires cognitive control of behaviour (e.g., anticipation and inhibition). Our aim was to explore the integrity of saccadic eye movement control in developmental coordination disorder (DCD) and typically developing (TD) children (8–12 years) and assess how these children plan and inhibit saccadic responses, the principal mechanisms within visual attention control. Eye movements and touch responses were measured (separately and concurrently) in Cued and Non-Cued conditions. We found that children with DCD had similar saccade kinematics to the TD group during saccade initiation. Advance information decreased hand movement duration in both groups during Cued trials, but decrements in accuracy were significantly worse in the DCD group. In addition, children with DCD exhibited greater inhibitory errors and inaccurate fixation during the Cued trials. Thus, children with DCD were reasonably proficient in executing saccades during reflexive (Non-Cued) conditions, but showed deficits in more complex control processes involving prediction and inhibition. These findings have implications for our understanding of motor control in children with DCD.

## Introduction

Motor impairment amongst children is a widespread problem. Estimates suggest that 5% of the population have some form of motor disorder that has long-term implications for physical and mental health [1]. Developmental coordination disorder (DCD) is a broad diagnostic construct encompassing heterogeneous presentations. It is a term used to describe children with a core motor deficit in the absence of overt signs of other conditions that might explain the motor difficulties. More specifically, according to the DSM-5 diagnostic criteria, DCD is determined when: a child presents impairment in the acquisition and learning of motor skills in comparison to peer groups (criteria A), these motor deficits significantly and persistently affect activities of daily living and impact academic achievement, leisure and play (criteria B), the onset of motor deficits occur early in development (criteria C), and the deficits cannot be explained by other intellectual disability, visual deficit or other neurological impairment, such as cerebral palsy (criteria D) [2]. In addition, many report the co-occurrence of social and affective problems in DCD, including a lack of concentration, general behavioural problems, poor social competence and poor participation in physical activities [3].

The aetiology of DCD is not well understood and a number of factors may influence the probability of a child meeting the diagnostic criteria (e.g. genetic deficits, birth trauma, etc.) [1]. Nevertheless, there have been numerous attempts to construct causal process-orientated hypotheses to explain the presence of the motor deficits. For example, Wilson and McKenzie [4] identified increased difficulties with ‘visual-spatial processing’ tasks within the DCD population. This review of 50 studies concluded that “perceptual problems, particularly in the visual modality, are associated with difficulties in motor coordination” [4]. The difficulty with such a conclusion is that it rests on the observations of how children have responded (using the motor system) to perceptual stimuli. There is no study to date that has established a perceptual system deficit per se as being a necessary or sufficient feature of DCD. In the absence of evidence for a perceptual deficit, the observation that children show problems in generating responses to perceptual stimuli could be due to the children having motor difficulties rather than a specific perceptual impairment.

More recently, it has been hypothesised that a fundamental deficit in the ability to utilise internal models may underlie the compromised motor control exhibited by children with DCD [5]. Internal models have been extensively used in explaining the control of actions in a number of adaptive behaviours such as reaching, walking, and eye movements [6]. These internal models estimate the sensory consequences of an action, prior to the use of feedback information, and when planning a motor response; they can thereby minimise sensory feedback delays [7, 8]. The internally-generated predictions (of sensory consequences) allow for more accurate estimations of the requisite motor signals to be formulated. It is suggested that this internally-generated model is key to the poor motor control observed in DCD [7–9].

The ‘internal model deficit’ hypothesis seems to be difficult to falsify, given that most movement control requires the use of internal models [10–12]. Moreover, reports suggest that children with DCD have similar saccadic eye movement control relative to TD children [13–15]. However, the observations that saccadic eye movements are equivalent in both DCD and TD children tends to be made with regard to simple responses to visually-guided targets [13–15]. Notably, children with DCD do appear to have difficulties in more complex saccadic tasks, such as generating double-step saccades [15], predicting target location [7] and when programming a coordinated (eye and hand) response versus generating a simple eye movement alone [13]. The apparent conflict between these findings (i.e. normal vs abnormal saccade control) can be examined by making direct comparisons between tasks that involve visually-guided reactive responses and actions that require higher order cognitive control, such as planned motor responses. Given the similarities in saccade kinematics between DCD and TD groups in visually-guided tasks, existing deficits in DCD may be associated with the cognitive (attention) control mechanisms of anticipation and inhibition rather than saccadic control per se. This hypothesis is in line with studies that show deficits in DCD during attentional shifts (as saccade latency and inhibition errors) and during volitional control of attention (for review see [5]). Attentional control mechanisms are critical for planning and responding to cued stimuli, and internally generated responses cannot be accurately formulated without this ability. These cognitive control deficits could explain why children with DCD fail at more complex tasks and struggle with the acquisition of new skills.

The present study investigated saccadic eye movements and hand movements in children with DCD and TD controls during cued and non-cued conditions. The cued condition used in this study utilizes both inhibition of a response and anticipation of the next target position (pre-programmed response) and thus may provide a useful indication of the balance achieved between these mechanisms in children with DCD [16]. The experiment was designed in order to: (i) test the hypothesis that children with DCD have difficulties in the cognitive control (inhibition-anticipation) required when planning a motor response (cued conditions), and (ii) determine whether deficits are specific to the coordination of the eye and hand as reported by Wilmut and colleagues [13]. To achieve this, we examined group differences in planning and executing an eye movement alone (EO), hand movement alone (HO) or during the coordination of both actions (EH). Deficits in planned responses due to cognitive (attention) control mechanisms were determined by examining fixation ability, inhibition errors, saccade latency, and the accuracy of the planned response in cued compared to non-cued conditions. In addition, comparing the difference between single versus coordinated responses was undertaken to provide insight into how cognitive control deficits might be manifest within this population.

## Methods

### Participants

The study recruited ten children in the age range of 8–11 years (mean = 10.1 ± 1.0 years; 3 females; 7 males) who met the DSM diagnostic criteria for DCD [2,17]. The children with DCD were a clinical population recruited from a private clinic offering intervention for their movement difficulties after being diagnosed with DCD (the diagnoses were made within an NHS clinic and the children were referred from the NHS clinic to the private treatment facility). The children with DCD were from the City of York and surrounding area. The diagnoses were made by qualified medical practitioners (i.e., a team including medical doctors and occupational therapists). In addition, twelve typically developing (TD) children were recruited from a local primary school in Leeds, UK. These children had no history of motor, ophthalmological or cognitive deficits (such as DCD or ADHD) and had an age range of 8–12 years (mean = 10 ± 1.12 years; 4 females; 8 males) to match the DCD population (see Table 1). None of the TD children had any history of motor deficits and this was validated by their parents and teachers who reported that the children were progressing well within home and school (allowing us confidence that these children were not at risk of DCD). None of the children (TD or DCD) had a diagnosis of ADHD and all participants were right-handed, based on self-reports on key indicators (e.g. writing, throwing, pointing, grasping). The child’s report of hand preference was confirmed by the parents as being the hand the child predominantly used during the majority of everyday tasks. No child or parent had difficulties identifying the right hand as the preferred hand.

**Table 1:** Participant’s characteristics (age and sex) across the DCD and TD groups.

DCD			TD		
Participant	Age (years)	Sex (male/female)	Participant	Age (years)	Sex (male/female)
1	10	m	1	11	f
2	11	f	2	9	f
3	11	m	3	10	m
4	9	m	4	9	m
5	10	f	5	8	m
6	10	m	6	10	m
7	11	m	7	11	m
8	8	f	8	12	m
9	10	m	9	10	f
10	11	m	10	11	m
			11	9	f
			12	10	m

### Ethics Statement

Informed written and oral consent was obtained from the child participant and their parent or guardian. All participants were informed that they could stop the experiment at any point during the session. The study was approved by the University of Leeds ethics committee and conducted in accordance with the ethical standards laid out in the 1964 Declaration of Helsinki and in accordance to the British Psychology Society (BPS).

### Experiment setup

**Fig 1:**
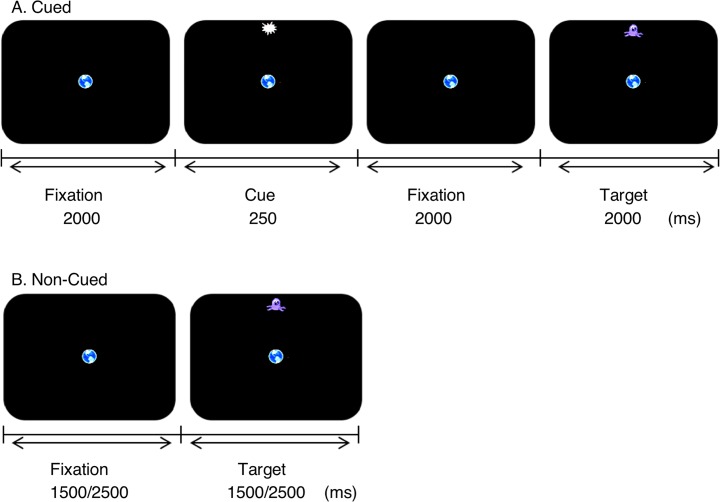
A) Cued and B) Non-Cued trials. Timings and target locations were predictable during the Cued condition, whilst target timings and locations were random in the Non-Cued condition. Targets (alien image) and cues (blast image) could appear at one of four locations from the centre fixation (earth image) point (90°, 180°, 270° and 360°). Central fixation was always present and the cue was always valid. Participants performed each condition with eyes only (EO), hand only (HO) and eyes and hand (EH). Schematic adapted from [16].

Participants were seated in a height adjustable saddle chair to improve postural stability and their heads were supported by a chin and forehead rest, to restrict head movements, 38 cm from a touch screen computer (19” colour monitor, 1024 by 768 pixel resolution, with a refresh rate of 85Hz, touch screen activation force of 50–120 grams per square centimetre and an accuracy that exceeds .3 cm, Elo Touch Solutions, Inc). All participants were assessed prior to the experiment to ensure they could view all targets presented on the screen and reach (with ease) with an elbow support provided between each reach movement to reduce fatigue. Touch responses were recorded (with the same resolution as the monitor) from the participant’s preferred hand, which was placed in front of the participants on a resting block. Eye movements were recorded using an eye-tracker sampling at 1000 Hz (Eyelink 1000, SR research, Canada) and a tower mounted camera in which the eye signal was not disrupted by the reaching movements. A separate computer recorded and stored the data for subsequent offline analysis. All visual stimuli were presented using Experiment Builder software (SR research, Canada). All objects presented (central fixation, cue and 4 targets) were 1 x 1 degrees of visual angle (°) or 50 pixels in diameter and presented on a black background (luminance of 50 cd/m2). To avoid confusion and engage the participant, each object presented differed and consisted of an image of earth, a blast or explosion and an alien as central fixation point, peripheral cue and targets respectively (see Fig 1, and for further details on the task also see [16]). Experimental sessions took place in a dark quiet room to avoid any distractions. Rests were provided between each experimental block and when needed and the lights were turned on during these rest periods to maintain alertness and minimize dark adaptations. Experimental sessions were kept under 60 min.

### Experiment Protocol

Each experimental block started with a nine-point calibration, followed by a validation of the eye position based on this initial calibration. Practice trials were introduced at the beginning of each block under close observation of the experimenter to ensure all participants were performing the tasks correctly. All participants received the same amount of practice and were able to verbally report what the requirements of the task were prior to commencing each experimental block. An eye drift correction was also implemented at the beginning of each experimental block, after practice trials, to avoid data loss.

Participants performed i) eye movements only (**EO**), ii) hand movements only (**HO**), whilst eyes were fixated on the centre of the screen, and iii) eye and hand movements together (**EH**) to targets presented on the screen during Cued and Non-Cued conditions. In the **Cued (C) condition**, a central fixation point was presented for 2000 ms, after which a cue was presented for 250 ms and 9° from the centre in one of four locations along the horizontal and vertical axis (at 90°, 180°, 270° and 360°). A target then appeared 2000 ms after the cue offset and remained visible for 2000 ms (Fig 1A). The cue was always valid (same location as the target) and all participants were asked to inhibit any type of response (eye and/or hand) to the cue and maintain fixation on the centre of the screen until the target appeared. For the **Non-Cued (NC) condition**, participants fixated a central target for 1500 or 2500 ms, after which a target appeared in one of the four locations. This target remained visible for either 1500 or 2500 ms. Fixation and target timings were randomized between (Non-Cued) trials (Fig 1B). The central fixation point was present throughout all the trials but disappeared with the target to signal the start of a new trial (inter-trial time of 1000 ms). Target and cue locations were randomised between experimental blocks and participants.

There were a total of six experimental blocks (C and NC x EO, HO and EH tasks) and each consisted of 32 trials. Participants were asked to respond to the target as fast and as accurately as possible with either their eyes only (EO), their hand only (HO) or both eye and hand together (EH). More explicitly, participant’s instructions were to “look and touch earth” (central fixation point) and then “look/touch” (while looking at earth)/ “look and touch the alien” (target) “as soon as it appears, as fast and as accurately as possible” in the Non-Cued condition; and were asked to “look and touch earth” (central fixation point), “don’t look or touch the blast” (cue) and “keep looking at earth” (central fixation); and then “look/touch” (while looking at earth)/”look and touch the alien” (target) “as soon as it appears, as fast and as accurately as possible” in the Cued condition. They were also told that the alien (target) would appear at the same location as the blast (cue) but that they should not respond to the blast (cue) and instead wait for the alien (target). The six conditions were blocked in the following order for the children to avoid confusion between tasks and potential task switching effects: Non-Cued EO, EH and HO; and Cued EO, EH and HO. In addition, the consistent ordering of blocks was necessary to ensure that we could compare between groups (as all children then completed exactly the same block/task order).

### Data analysis

#### Eye movements

Participants’ eye movement data were obtained from the Data Viewer software (SR research, Canada). Blinks were automatically eliminated from the data before analysis. Eye and hand data were divided into “events” consisting of fixation, cue and target presentation. In addition, areas of interest (AOI) were determined for the centre fixation and the four possible target centres (each AOI forming a 200 x 200 pixel box, equivalent to 3° distance around the centre point of each stimulus). Initially, all saccades (>2°, to exclude fixational eye movements) were extracted across the whole trial and used for the main sequence analysis. This resulted in a broader range of saccadic amplitudes allowing a comparison between saccade amplitude and duration across our populations. This was followed by a more detailed analysis where viable saccades were identified as samples with a minimum velocity of 100°/s and within 3° of the target area. The corresponding saccade latency, alongside the saccade end locations in X and Y coordinates were computed and compared with the actual target location to obtain absolute error (i.e., the absolute distance of the eye location to the centre of the target). Anticipatory saccades (< 80ms from target onset) were expected during Cued conditions and included in the saccade latency analysis [16]. To identify deficiencies in saccade generation, the saccade amplitude/duration relationship or ‘main sequence’ [18] (typically linear at < 50°) (of all saccades > 2°) was calculated using regression analysis. To identify attention (inhibitory) control, the number of saccades made during central fixation and cue presentation was identified and reported as a mean ratio between the number of saccades observed per trial and the number of total trials per condition.

#### Hand responses

The timing and location of the touch responses were obtained together with the eye data from the Data Viewer software. Touch time was defined as the time from target onset until the target was touched on the computer’s screen and thus included both reaction time and movement time. Accuracy was measured as the absolute error from the target’s (centre) position.

Eye and hand data were fed into a multivariate design using a repeated measures analysis of variance (ANOVA) (SPSS version 20, IBM, USA) for each parameter (latency and accuracy). Interactions between variables were evaluated using a Bonferroni corrected post-hoc test. Non-parametric tests were performed due to breaches in normality, particularly in the ‘number of saccades’ data. Kruskal-Wallis and further Wilcoxon tests were performed to identify differences between groups and within experimental blocks. A significance level of *p* < .05 was established for all statistical analyses. Effect sizes are reported as partial eta squared *η*_*p*_^2^ values and Cohen’s *d*, as well as r values when appropriate. All results and graphs are expressed as means and standard error of the mean (SEM). Significant differences between conditions and groups are highlighted in all graphs using * and † symbols.

## Results

For clarity, we separated eye and hand results. Eye responses were examined to address our aim of investigating cognitive (attention) deficits between the groups and how these deficits, if present, affect the planning of an eye response, a hand response or uniquely a coordinated eye and hand response (our second aim). We first examined saccade kinematics (main sequence) in both groups to identify any underlying deficiencies in the generation of saccades, which could account for any accuracy or latency differences seen between the groups in our experimental conditions. We then examined eye responses obtained from the EO and EH tasks in both Cued and Non-Cued conditions. In addition, since eye movements can behave differently with the inclusion of a coordinated hand or arm movement, we compared saccade kinematics (latencies and accuracies) between these EO and EH tasks. Errors (inhibition) during fixation and cue presentation were measured; also changes in saccade latencies and accuracies during target presentations in Cued (planned responses) versus Non-Cued conditions were obtained for both groups. Finally, hand responses to the target were then analysed from the HO and EH tasks in both cue conditions.

### Eye movements

#### Saccade kinematics: main sequence

**Fig 2:**
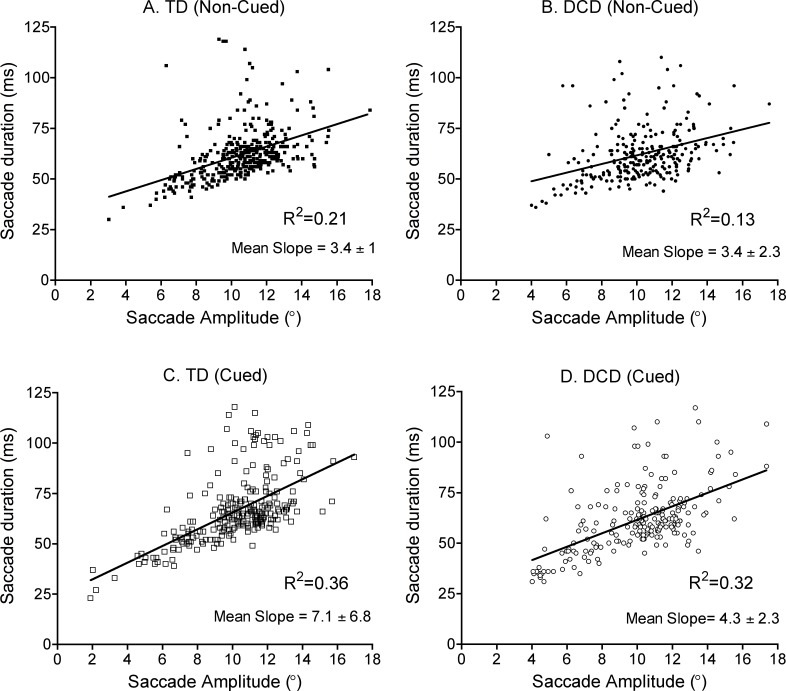
The reported data are for all saccades made within the trial that exceeded 2° in amplitude during the eye only (EO) task. Saccade duration and amplitude (main sequence) of TD children (A and C, left) and children with DCD (B and C, right) during Non-Cued (A and B, top graphs) and Cued (C and D, lower graphs) conditions. The graph shows the relationship between the saccade’s duration and corresponding amplitude. These included anticipatory saccades from the Cued conditions as these occurred in more than 50% of trials in the DCD group. All graphs show significant R^2^ values, which reveal a linear relationship between these saccade parameters in both groups and show the mean slopes obtained from each participant’s main sequence. Comparisons between group and condition did not reveal significant differences.

To explore saccadic kinematics in both the DCD and TD groups, a “main sequence” regression was performed on all saccades made within a trial (> 2°), during fixation onset, cue onset, cue offset and target onset (including saccades to the cue, anticipatory saccades to target location, saccades back to centre fixation). Regressions of saccade amplitude and duration were found to be significantly correlated (R^2^ = .21, F(1,369) = 94, *p* < .001 for TD and R^2^ = .13, F(1,330) = 39, *p* < .001 for DCD groups during Non-Cued conditions; and R^2^ = .36, F(1,346) = 140, *p* < .001 for TD and R^2^ = .32, F(1,313) = 98, *p* < .001 for DCD groups during Cued conditions). Both groups show a linear relationship between saccade duration and amplitude (Fig 2). Slope differences between Cued and Non-Cued conditions indicate the existence of anticipatory saccades, which are typically smaller in amplitude [19], but still within the main sequence linear relationship. A further repeated measures ANOVA of the individual main sequence slopes was performed and did not show significant differences between groups (*p* = .38) or conditions (*p* = .07) (Fig 2).

#### Fixation and inhibition errors

**Fig 3:**
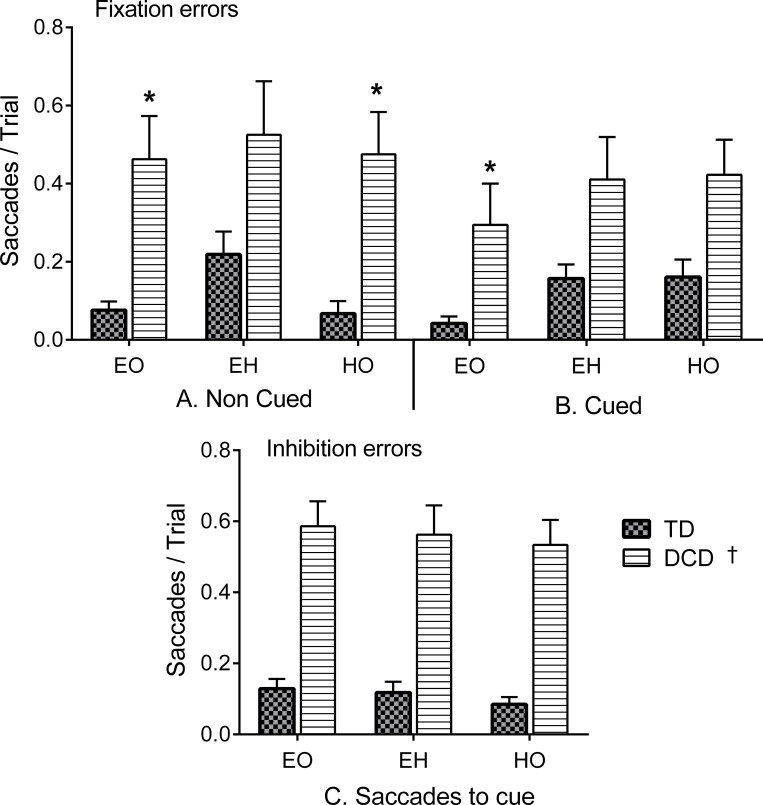
Mean (and SEM) number of saccades per trial ratios for Non-Cued (A, left) and Cued (B, right) across EO, EH and HO blocks. The graph shows the ratio of larger saccades away from fixation, which was higher in the DCD compared to the TD group (DCD vs. TD, *). **Lower panel.** Mean number of saccades per trial (and SEM) during the presentation of the cue (250 ms) across Cued (EO, EH and HO) conditions (C). Overall, children with DCD exhibited greater number of saccades and thus, more inhibition errors compared to the TD group (DCD vs. TD, †).

Participants were aware that a target or a cue would appear within the vicinity, thus we tested whether the children with DCD were able to maintain fixation on the centre target compared to the TD group. For this, we computed the number of saccades away from fixation that exceeded a 3° box around the fixation target during the fixation period. The data did not conform to a normal distribution so a non-parametric test was applied to these data with a more stringent p value of p < .01. These non-parametric tests revealed that the children with DCD exhibited more saccades away from fixation compared to TD children in EO Cued conditions (χ2 (1) = 9.9, *p* = .002) and in EO and HO Non-Cued conditions (χ2 (1) = 8.7, *p* = .003 and χ2 (1) = 13.31, *p* < .001 respectively) (Fig 3A and 3B) but errors did not reach statistical significance (*p* < 0.01) in EH and HO Cued conditions (χ2 (1) = 4.28, *p* = .038 and χ2 (1) = 6.14, *p* = .013 respectively) or EH Non-Cued conditions (χ2 (1) = 4.13, *p* = .042). Post-hoc tests showed that only the TD children exhibited increases in the number of saccades in EH compared to EO in the Cued conditions (Z = -2.99, *p* = .003, r = -.86 for EO vs. EH). A marginal increase was also observed in HO, but errors did not reach statistical significance (Z = - 2.57, *p* = .010, r = -.74 for EO vs. HO).

To further test the group’s inhibitory control, we examined whether the children made saccades away from the fixation point and to the cue during Cued conditions. The number of saccades made (inhibition errors) was computed as a mean ratio of the number of saccades per trial. Non-parametric analysis revealed that the children with DCD exhibited more saccades to the cue (inhibition errors) compared to the TD group across all tasks (χ2 (1) = 16.5, *p* < .001, χ2 (1) = 15.74, *p* < .001, χ2 (1) = 14.87, *p* < .001 for EO, EH and HO respectively) (Fig 3C).

#### EO and EH responses to the target: visually-guided vs. planned saccades

**Fig 4:**
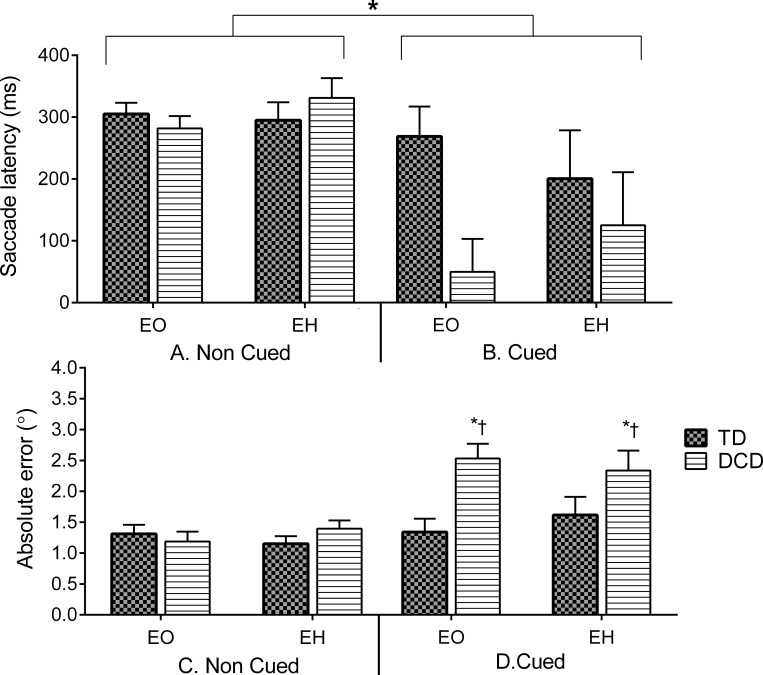
Mean (and SEM) Non-Cued (A) and Cued (B) saccade latencies across eye only (EO) and eye and hand (EH) tasks. Overall, the groups decreased saccade latencies in Cued conditions (Cued vs. Non-Cued, *). **Lower panel.** Mean Non-Cued (C) and Cued (D) saccade accuracy (and SEM) across eye only (EO) eye and hand (EH) tasks. Children with DCD (striped columns) exhibited increased absolute errors in the Cued conditions compared to the TD children (dark grey, D) and compared to the Non-Cued conditions (group by cue condition interaction, *†). However, there were no accuracy or latency differences between the groups in the Non-Cued conditions (C).

We measured the latency and the accuracy (absolute error) of the first saccade to the target (within the target’s area) during EO and EH tasks for both Cued and No-Cued conditions. These responses included anticipatory saccades as described in the methods section. We compared whether eye movements differed when adding the hand in the EH task and then compared between cue conditions. Saccade latency analysis did not reveal significant differences between the groups in the different EO and EH tasks (*p* > .05). Saccadic latencies for both groups differed only between conditions with Cued latencies being shorter than Non-Cued (cued condition effect *F*(1,20) = 11.17, *p* = .003, *η*_*p*_^2^ = .37) (Fig 4A and 4B). Group interactions with Cued conditions did not reach statistical significance (*p* = .08).

Saccade accuracy to target revealed a significant group by cue condition interaction (*F*(1,20) = 8.79, *p* = .008, *η*_*p*_^2^ = .289) (Fig 4C and 4D). Post hoc tests revealed that the children with DCD made larger errors (relative to the target) when compared to the TD children in the Cued conditions (*p* = .005, *d* = .83). The children with DCD revealed increased error to the cued targets with many of the saccades being anticipatory in nature. This increase in error to the target has been reported previously in healthy adult populations [19]. In addition, these errors were greater compared to the DCD group’s visually guided responses in the Non-Cued condition (*p* < .001, *d* = .39). The children with DCD exhibited anticipatory saccades in over 50% of trials, compared to a mean of 15% of trials in TD children.

### Hand responses

#### HO and EH responses to the target: visually-guided vs. planned touch responses

**Fig 5:**
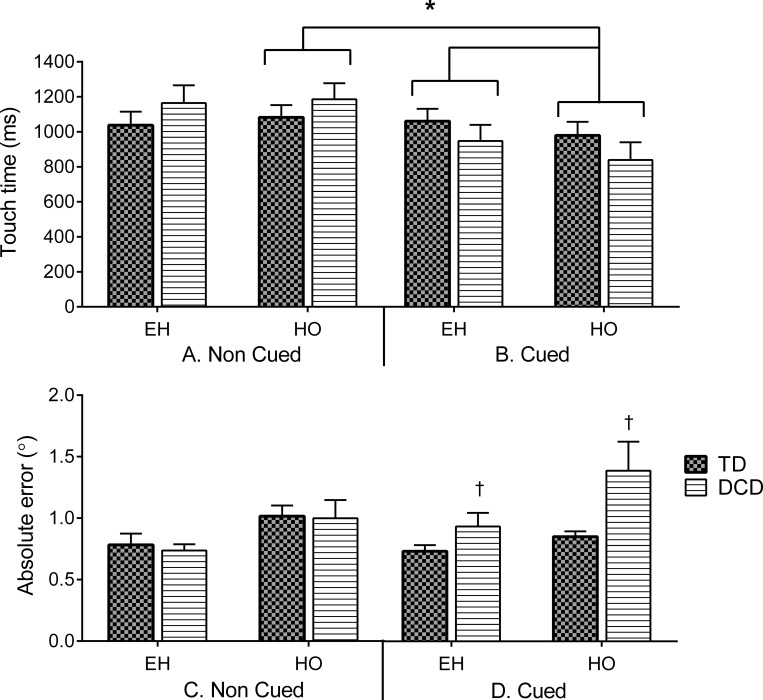
Mean Non-Cued (A) and Cued (B) touch times (and SEM) from target onset across coordinated (EH) and hand only (HO) tasks. Both groups decreased HO touch times in the Cued compared to the Non-Cued conditions and compared to EH (Cued HO, *). Decreases in touch time seemed to be more evident in the DCD group (~ 343 ms difference) compared to the TD group (~ 100 ms difference), however, this result did not reach statistical significance. **Lower panel**. Mean Non-Cued (C) and Cued (D) touch accuracy (as mean absolute error and SEM) across EH and HO tasks. Children with DCD (striped columns) were less accurate compared to TD children (grey columns) in Cued (D) conditions (DCD vs. TD, †).

Touch times and accuracies were obtained from the Cued and Non-Cued EH and HO blocks. Touch times of hand responses (Fig 5A and 5B) only revealed significant interactions for the different experimental conditions and tasks (cue conditions x EH and HO tasks, *F*(1,20) = 6.82, *p* = .018, *η*_*p*_^2^ = .29) with both groups able to decrease their HO touch time during Cued compared to Non-Cued conditions (*p* = .004, *d* = .84). Furthermore, touch times between EH and HO tasks were significantly different in the Cued condition, with shorter HO durations compared to EH (*p* = .04, *d* = .37) and no statistically significant group interaction (*p* = .09).

A significant task effect (*F*(1,20) = 34.19, *p* < .001, *η*_*p*_^2^ = .67) revealed that participants were more accurate when touching the target in EH than when eyes were fixed, but a task by group interaction was not significant (*p* = .61). In addition, a cued condition x group interaction (*F*(1,20) = 4.66, *p* = .037, *η*_*p*_^2^ = .21) showed that the TD children were more accurate than the DCD group in the Cued condition (*p* = .013, *d* = .81)(Fig 5C and 5D).

## Discussion

We found similarities in the responses of children with and without DCD to visually-triggered (Non-Cued) targets in both eye and hand movements. This suggests both groups have no or small problems with ‘visual-spatial processing’, meaning that our original hypothesis (as presented in the introduction) could not be rejected. A more detailed exploration of the saccade kinematics (main sequence) revealed that the children with DCD were generating saccades that did not differ from the TD group’s main sequence relationship, suggesting that they possess uncompromised basal ganglia and brainstem function and the requisite internal models (controllers) for these movements [11,20,21]. Wilmut and Wann [7] also found that children with DCD have similar eye latencies and accuracy when compared to TD children during visually-guided (non-cued) conditions.

In contrast, differences did exist in the Cued condition. Specifically, children with DCD showed a greater number of saccades away from fixation in the Cued condition (inhibition) and greater number of anticipatory saccades following the provision of advance cue information (before presentation of the stimulus), indicating poor inhibitory control. In addition, children with DCD showed larger errors in accurately localizing the remembered location of the target compared to the TD group. This is again consistent with our original hypothesis that inhibition mechanisms, vital for saccadic control, are compromised in children with DCD and that deficits in cognitive control may affect the planning and execution of a complex response.

It is clear that the increased anticipatory responses in the DCD population, compared to TD children, did not result in enhanced touch performance, as shown by the lower touch accuracy. It was also interesting to note that the children with DCD did not slow down their responses to increase this accuracy, suggesting that they prioritised speed over accuracy. Faster touch times to the target were accompanied by poor inhibitory control in the DCD groups as these children were making saccades in both HO and EH tasks during the cue presentation, which is in agreement with findings suggesting a “look-then-move” behaviour [14]. Supporting this, the different behavioural results between the EH and HO tasks (faster touch times but reduced touch accuracy) suggests that moving the eye to the target significantly benefits the DCD group. We suggest that the peripheral visual information provided in the HO task was not used as effectively in children with DCD when compared to the TD group, perhaps due to failures in inhibitory control mechanisms, resulting in poor visual spatial acquisition during the cue presentation.

Our findings are in agreement with previous studies that have found group differences in children with DCD during volitional shifts of attention, and associated deficits in manual and oculomotor inhibitory control [22,23]. Mandich et al [23] proposed that “inhibition differences between DCD and control children are particularly evident when the to-be-supressed response is provoked by an external stimulus”. The errors in inhibition occurred regardless of the motor effector recruited (eye only, eye and hand or hand only), indicating that deficits in control are not solely related to tasks that require the integration of visual information with other motor systems (i.e. during coordination) [13]. Fixational errors also support these inhibition deficits in the DCD group, albeit these volitional responses likely reflect a high expectancy of the upcoming stimulus (anticipation) and not a stimulus-driven response.

The picture that emerges from these studies is consistent with the idea that children with DCD have difficulties with the cognitive control processes that balance inhibition and anticipation. The current study shows that the inability to inhibit a saccadic movement to a cue (a function associated with the frontal eye fields, [24]) results in inappropriate allocation of visual attention. Conversely, the impaired attentional capabilities observed in the DCD population will interfere with the children’s ability to generate skilled hand movements. This in turn will hinder the development of skilled hand movements.

The results within this manuscript raise the issue of the source of the difficulties with balancing inhibition and anticipation in saccadic control. Only a few studies have investigated the neurological deficits in DCD, but those studies that do exist suggest disruption to the fronto-parietal [25] and cerebellar [26] networks. Notably, the fronto-parietal network is important for short-term memory and anticipation and has a general ‘attentional’ function [27]. The frontal and supplementary eye fields together with the dorsolateral prefrontal cortex have been associated with saccade inhibition and predictive mechanisms [28–30]. Furthermore, Gonzalez et al. [16] showed differences in the saccadic balance between inhibition and anticipation as a function of age. The developmental problems found here suggest that interference within the fronto-parietal and cerebellar networks may disrupt the development of a higher order ‘control’ network important for saccade inhibition. This disruption might be sufficient to interfere with the control mechanisms that support the allocation of visual attention–creating deficits in the control of saccadic eye movements and, in consequence, the acquisition of numerous skilled behaviours that require accurate visual attention.

Our suggestions are consistent with previous studies which have shown deficits in both ventral (externally driven) and dorsal (top-down) attentional networks in DCD [22,23]. Our experimental task required both control systems to suppress saccades to the advance cue (bottom-up, reflexive) and respond to the target at the appropriate time (top-down, volitional). The neural networks involved in eye movements play a central role in the allocation of visual attention [31], thus disruption to saccadic control is likely to have a detrimental effect on skilled behaviors that rely on the rapid acquisition of visual information within the environment (e.g. bimanual aiming movements). The outcome of our study would suggest that interventions that target optimizing the balance between inhibitory control and anticipation mechanisms (such as pursuit eye movements) might prove beneficial to children with DCD.

It is interesting to note that studies involving children with ADHD have shown similar findings to the present study, with the ADHD population showing saccade metrics similar to those of TD controls, but showing problems with inhibitory control [32]. The children with DCD in the present study did not have a formal diagnosis of ADHD which may in large part reflect the fact that their motor difficulties were overt and extreme. In complex diagnostic constructs (such as DCD and ADHD), it becomes particularly difficult to identify the core difficulties experienced by the children. However, the similarity between the eye movements found in this DCD population and previous studies of children with ADHD [32] raises the question of the extent to which these developmental disorders overlap, and also highlights the broadness of the DCD diagnosis which includes potential co-morbidities. As such, a limitation of the current study is in knowing whether the deficits observed are related to a co-occurrence of sub-clinical ADHD in our DCD population. Very few studies on DCD have implemented an ADHD exclusion in their clinical group, however those that have, including Tsai and colleagues [22] and Wilson et al., [4] suggest that observed attentional deficits are specific to DCD and not the consequence of symptoms of ADHD. However, the commonality of co-morbidity of DCD with ADHD requires further study in this population.

The difficulties with saccadic control evidenced by the children had a direct impact on their overt allocation of visual attention (by definition), showing the tight coupling between the broad and highly related constructs of ‘motor skill’ and ‘attention’. Our study included a small group with a narrow age range in which age-related cognitive control of attention could be compared to a TD group. Whether these deficits are present across the range of motor abilities within the DCD population is unknown and warrants further investigation. In addition, longitudinal approaches and comparisons between larger groups of participants (with a wider age range) may show how these deficits develop in DCD and provide further insights into rehabilitation. Nevertheless, the identification of problems with saccadic control sheds some light on some potential underlying difficulties within the DCD population at the neural level, but care must be taken in mapping these deficits to behavioural outcomes (and vice versa). We suggest that at a clinical level, the most important step is identifying those problems which provide the greatest barriers to critical activities of daily living and supporting the child in overcoming these hurdles.

## Conclusion

We explored the ability of children with DCD to plan and inhibit their saccadic responses to visual targets. We found that saccade generation was equivalent in both TD and DCD groups, but failures in the balance between inhibition and anticipation in children with DCD resulted in decreased performance relative to TD children. The key role played by saccadic control in the allocation of visual attention suggests that these deficits could make it difficult for children with DCD to acquire complex skills. The findings also suggest interventions that might help to optimise this inhibition–training inhibition control is one such possibility.

## References

[1] Kirby A, Sugden D, Purcell C. Diagnosing developmental coordination disorders. Arch Dis Child. 2014;99: 292–296. 10.1136/archdischild-2012-303569 24255567

[2] American Psychiatric Association. Diagnostic and Statistical Manual of Mental Disorders [Internet]. Fifth Edition American Psychiatric Association; 2013 Available: http://psychiatryonline.org/doi/book/10.1176/appi.books.9780890425596

[3] Losse A, Henderson SE, Elliman D, Hall D, Knight E, Jongmans M. Clumsiness in children—do they grow out of it? A 10-year follow-up study. Dev Med Child Neurol. 1991;33: 55–68. 1704864 10.1111/j.1469-8749.1991.tb14785.x

[4] Wilson PH, McKenzie BE. Information processing deficits associated with developmental coordination disorder: a meta-analysis of research findings. J Child Psychol Psychiatry. 1998;39: 829–840. 9758192

[5] Adams ILJ, Lust JM, Wilson PH, Steenbergen B. Compromised motor control in children with DCD: a deficit in the internal model?—A systematic review. Neurosci Biobehav Rev. 2014;47: 225–244. 10.1016/j.neubiorev.2014.08.011 25193246

[6] Wolpert DM, Ghahramani Z, Jordan MI. An internal model for sensorimotor integration. Science. 1995;269: 1880–1882. 7569931 10.1126/science.7569931

[7] Wilmut K, Wann J. The use of predictive information is impaired in the actions of children and young adults with Developmental Coordination Disorder. Exp Brain Res. 2008;191: 403–418. 10.1007/s00221-008-1532-4 18709366

[8] Zwicker JG, Missiuna C, Harris SR, Boyd LA. Brain activation associated with motor skill practice in children with developmental coordination disorder: an fMRI study. Int J Dev Neurosci Off J Int Soc Dev Neurosci. 2011;29: 145–152. 10.1016/j.ijdevneu.2010.12.002 21145385

[9] Zwicker JG, Missiuna C, Boyd LA. Neural correlates of developmental coordination disorder: a review of hypotheses. J Child Neurol. 2009;24: 1273–1281. 10.1177/0883073809333537 19687388

[10] Robinson DA. Models of the saccadic eye movement control system. Kybernetik. 1973;14: 71–83. 10.1007/BF00288906 4206845

[11] Chen-Harris H, Joiner WM, Ethier V, Zee DS, Shadmehr R. Adaptive Control of Saccades via Internal Feedback. J Neurosci. 2008;28: 2804–2813. 10.1523/JNEUROSCI.5300-07.2008 18337410 PMC2733833

[12] Quaia C, Lefèvre P, Optican LM. Model of the control of saccades by superior colliculus and cerebellum. J Neurophysiol. 1999;82: 999–1018. 10444693 10.1152/jn.1999.82.2.999

[13] Wilmut K, Brown JH, Wann JP. Attention disengagement in children with developmental coordination disorder. Disabil Rehabil. 2007;29: 47–55. 10.1080/09638280600947765 17364756

[14] Wilmut K, Wann JP, Brown JH. Problems in the coupling of eye and hand in the sequential movements of children with Developmental Coordination Disorder. Child Care Health Dev. 2006;32: 665–678. 10.1111/j.1365-2214.2006.00678.x 17018042

[15] Katschmarsky S, Cairney S, Maruff P, Wilson PH, Currie J. The ability to execute saccades on the basis of efference copy: impairments in double-step saccade performance in children with developmental co-ordination disorder. Exp Brain Res. 2001;136: 73–78. 11204415 10.1007/s002210000535

[16] Gonzalez CC, Mon-Williams M, Burke MR. Children and older adults exhibit distinct sub-optimal cost-benefit functions when preparing to move their eyes and hands. PloS One. 2015;10: e0117783 10.1371/journal.pone.0117783 25659134 PMC4320084

[17] Black DW, Grant JE. DSM-5® Guidebook: The Essential Companion to the Diagnostic and Statistical Manual of Mental Disorders. American Psychiatric Pub; 2014.

[18] Bahill AT, Bahill KA, Clark MR, Stark L. Closely spaced saccades. Invest Ophthalmol. 1975;14: 317–321. 1123288

[19] Bronstein AM, Kennard C. Predictive eye saccades are different from visually triggered saccades. Vision Res. 1987;27: 517–20. 3660613 10.1016/0042-6989(87)90037-x

[20] Hikosaka O, Takikawa Y, Kawagoe R. Role of the basal ganglia in the control of purposive saccadic eye movements. Physiol Rev. 2000;80: 953–78. 10893428 10.1152/physrev.2000.80.3.953

[21] Sparks D, Rohrer WH, Zhang Y. The role of the superior colliculus in saccade initiation: a study of express saccades and the gap effect. Vision Res. 2000;40: 2763–77. 10960650 10.1016/s0042-6989(00)00133-4

[22] Tsai C-L, Pan C-Y, Chang Y-K, Wang C-H, Tseng K-D. Deficits of visuospatial attention with reflexive orienting induced by eye-gazed cues in children with developmental coordination disorder in the lower extremities: an event-related potential study. Res Dev Disabil. 2010;31: 642–655. 10.1016/j.ridd.2010.01.003 20163934

[23] Mandich A, Buckolz E, Polatajko H. On the ability of children with developmental coordination disorder (DCD) to inhibit response initiation: the simon effect. Brain Cogn. 2002;50: 150–162. 12372361 10.1016/s0278-2626(02)00020-9

[24] Izawa Y, Suzuki H, Shinoda Y. Response properties of fixation neurons and their location in the frontal eye field in the monkey. J Neurophysiol. 2009;102: 2410–2422. 10.1152/jn.00234.2009 19675294

[25] Alahyane N, Brien DC, Coe BC, Stroman PW, Munoz DP. Developmental improvements in voluntary control of behavior: effect of preparation in the fronto-parietal network? NeuroImage. 2014;98: 103–117. 10.1016/j.neuroimage.2014.03.008 24642280

[26] Zwicker JG, Missiuna C, Harris SR, Boyd LA. Developmental coordination disorder: a review and update. Eur J Paediatr Neurol EJPN Off J Eur Paediatr Neurol Soc. 2012;16: 573–581. 10.1016/j.ejpn.2012.05.005 22705270

[27] Majerus S, Cowan N, Péters F, Van Calster L, Phillips C, Schrouff J. Cross-Modal Decoding of Neural Patterns Associated with Working Memory: Evidence for Attention-Based Accounts of Working Memory. Cereb Cortex N Y N 1991. 2014; 10.1093/cercor/bhu189 25146374 PMC4717284

[28] Burke MR, Barnes GR. Brain and behavior: a task-dependent eye movement study. Cereb Cortex. 2008;18: 126–35. 10.1093/cercor/bhm038 17470446

[29] Connolly JD, Goodale MA, Goltz HC, Munoz DP. fMRI activation in the human frontal eye field is correlated with saccadic reaction time. J Neurophysiol. 2005;94: 605–611. 10.1152/jn.00830.2004 15590732

[30] Munoz DP, Everling S. Look away: the anti-saccade task and the voluntary control of eye movement. Nat Rev Neurosci. 2004;5: 218–228. 10.1038/nrn1345 14976521

[31] Corbetta M, Akbudak E, Conturo TE, Snyder AZ, Ollinger JM, Drury HA, et al A common network of functional areas for attention and eye movements. Neuron. 1998;21: 761–773. 9808463 10.1016/s0896-6273(00)80593-0

[32] Munoz DP, Armstrong IT, Hampton KA, Moore KD. Altered control of visual fixation and saccadic eye movements in attention-deficit hyperactivity disorder. J Neurophysiol. 2003;90: 503–514. 10.1152/jn.00192.2003 12672781

